# Hypercoagulability and the risk of recurrence in young women with myocardial infarction or ischaemic stroke: a cohort study

**DOI:** 10.1186/s12872-019-1040-4

**Published:** 2019-03-07

**Authors:** Alberto Maino, Ale Algra, Flora Peyvandi, Frits Richard Rosendaal, Bob Siegerink

**Affiliations:** 1 Department of Clinical Epidemiology, Leiden University Medical Center, Leiden, The Netherlands; 2 Angelo Bianchi Bonomi Haemophilia and Thrombosis Centre, Fondazione IRCCS Ca’ Granda Ospedale Maggiore Policlinico Milano, Università degli Studi di Milano, Milan, Italy; 3 Department of Internal Medicine, Azienda Provinciale per i Servizi Sanitari, Trento, Italy; 4 Department of Neurology and Neurosurgery, Brain Center Rudolph Magnus, University Medical Center Utrecht, Utrecht, The Netherlands; 5 Julius Center for Health Sciences and Primary Care, University Medical Center Utrecht, Utrecht, The Netherlands; 6 Department of Thrombosis and Haemostasis, Leiden University Medical Center, Leiden, The Netherlands; 7 Center for Stroke research Berlin, Charité Universitätsmedizin, Center for Stroke Research, Chariteplatz 1, 10117 Berlin, Germany

**Keywords:** Thrombosis, Myocardial infarction, Stroke in young adults, Prognosis, Recurrences

## Abstract

**Background:**

We aimed to investigate the role of hypercoagulability on the risk of lifetime cardiovascular recurrences after myocardial infarction or ischaemic stroke.

**Methods:**

Young women (< 50 years) with either myocardial infarction (*n* = 197) or ischaemic stroke (*n* = 107) were followed between 1995 and 2012 in the RATIO follow-up study. To determine whether hypercoagulability affects the risk or recurrence, a coagulation score based on acquired and inherited markers was compiled and used in a quartile analysis. Hazard ratios (HRs) obtained from Cox proportional models and adjusted for several cardiovascular risk factors were used to compare quartiles of the coagulation score for the risk of recurrence.

**Results:**

During a median follow-up of 19 years, 59 cardiovascular recurrences occurred. In patients with myocardial infarction no association was found between a high prothrombotic score and recurrences (highest quartile vs lowest quartile HR 0.7, 95% CI, 0.3–1.8). Conversely, ischaemic stroke patients with a high prothrombotic score showed a doubling in risk of long-term cardiovascular recurrences (HR 1.9, 95% CI 0.6–6.3) compared with ischaemic stroke patients and low levels of the score, with a dose response relationship.

**Conclusions:**

An increased coagulation tendency might be associated with long-term cardiovascular risk in women with ischaemic stroke, but not in women with myocardial infarction.

## Background

Young survivors of an arterial thrombotic event have an increased mortality and morbidity compared with the general population, mainly related to the high risk of cardiovascular recurrences [1–3]. This is particularly important at a young age, because the impact on quality of life and on socioeconomic costs, considering life expectancy, is the highest [4, 5]. Therefore, there is an increasing interest around secondary prevention strategies in the young to target risk factors for recurrences after a myocardial infarction or an ischaemic stroke event, the two main forms of arterial thrombosis.

Previous studies have shown that classical risk factors for the first episode, such as hypertension and hyperlipidaemia, are not associated with recurrences in both stroke and myocardial infarction [3, 6]. It is well known that the risk profile of a recurrent event can be different from that of a first event and that often a risk factor is found to be weaker for a second event than for a first [7, 8].

For the first cardiovascular event, we showed that an increased clotting propensity is a risk factor for ischaemic stroke, whereas the risk of myocardial infarction is only affected marginally [9–11]. No data are available on the association between hypercoagulability and a second arterial thrombotic event. The difference in the role of coagulation in the aetiology of a first occurrence of myocardial infarction and ischaemic stroke might also be relevant for the recurrence and prevention of these two diseases, and raises the question whether secondary prevention for ischaemic stroke and myocardial infarction should differ more than it does now.

Therefore, we investigated the role of an increased coagulation propensity on the risk of recurrent arterial thrombotic events in young women who survived myocardial infarction or ischaemic stroke.

## Methods

### Patients

We used data from the Risk of Arterial Thrombosis in Relation to Oral Contraceptives (RATIO) follow-up study, based on subjects who participated in the RATIO case-control study [12]. For the present study two groups of patients, subjects who survived either a myocardial infarction or an ischaemic stroke, were included. Patient selection within the RATIO case-control study has been described in detail previously [13–15]. In summary, the RATIO study included young women (between 18 and 49 years old) with either a first myocardial infarction or ischaemic stroke (index events) who consecutively presented to one of the 16 participating hospitals in the Netherlands between 1990 and 1995. Exclusion criteria were TIA (defined as an event lasting < 24 h), haemorrhagic stroke, venous sinus thrombosis, carotid artery dissection, history of cardiovascular or cerebrovascular diseases, severe illness, aphasia, or cognitive impairment interfering with the questionnaire and not speaking Dutch. Myocardial infarction was diagnosed by the presence of clinical symptoms, elevated cardiac enzyme levels, and corresponding electrocardiographic changes. Clinical symptoms of ischaemic stroke were confirmed by either computed tomography or magnetic resonance imaging. Ischaemic stroke of cardioembolic origin was excluded by the presence of atrial fibrillation or suggestive cardiac ultrasound imaging. All cases were included from 1995 to 1998 [16]. The population-based group of control women was identified by random digit dialing and matched according to age, area of residence, and year of event. Eligible as controls were women without prior myocardial infarction and ischaemic stroke, who were aged 18 to 49 years and who did not meet the exclusion criteria that were used for selecting patients. All participants were asked to fill in the same structured questionnaire comprising questions on medical history of cardiovascular risk factors. Smoking was defined as having regularly smoked in the year before the index date. Alcohol consumption was defined as drinking at least one unit of alcohol per week. Body mass index (BMI) was calculated as body weight (kg) divided by height squared (m^2^). Women were classified as hypertensive, diabetic or hypercholesterolaemic when they reported a physician’s diagnosis or were taking medication for these conditions before the index date. Family history of cardiovascular disease was defined as the presence of myocardial infarction, stroke or peripheral artery disease below 60 years of age in first degree relatives.

Blood samples and buccal swabs were collected for blood measurement of clotting factors and DNA analyses in a subset of women who initially participated in the first phase of the RATIO study (205 participants with myocardial infarction, 125 with ischaemic stroke and 638 controls). To compensate for the loss of statistical power in the ischaemic stroke group, we additionally recruited 50 women between 1996 and 2001, leading to a small difference in median time between event and blood sampling between the two case groups (69 months, range 38 to 117 months, for myocardial infarction cases and 95 months, range 23 to 146 months, for ischaemic stroke cases). The interval between the initial event and blood sampling minimises the risk of reverse causation as well as the mixing of short and long-term effects of hypercoagulability on cardiovascular risk. Blood draw procedures and laboratory procedures are described in detail in the previous publications [13–15]. All participants gave informed consent and the study was approved by the ethics committees of the participating hospitals [13].

In 2013, data on participants of the RATIO case-control study were linked to the Dutch Register of death certificates and to the Dutch Hospital Data register by the Central Bureau of Statistics of the Netherlands. The first provides both primary and secondary causes of deaths coded according to the International Statistical Classification of Diseases and Related Health Problems 10th Revision (ICD-10) classification. The second provides electronic coverage of data on all hospital admissions in the Netherlands since 1995. The data collected concern the date of admission and discharge, diagnoses, and surgical procedures from virtually all university and general hospitals and most specialized clinics. Diagnosis are encoded according to ICD 9th Revision (ICD-9). Almost all personal (age, sex, date of birth and postal code) and administrative data (date of admission, discharge and death), and 84% of principal diagnoses were found to be correctly encoded by a previous comparison of a random sample of hospital admissions in the Dutch Hospital Data register with information from hospital records [17]. For myocardial infarction, the percentage of correctly encoded diagnosis has since been found to be almost 100% [18].

Data from the RATIO case-control study were linked to these registries through date of birth, sex, and postal code of each RATIO participant. Individuals with information leading to more than one person (e.g., twins or individuals with the same date of birth in the same postal area) or to nobody at all, were excluded from the analyses. The original RATIO case control study included 1376 women (248 myocardial infarction cases, 203 ischaemic stroke cases and 925 controls). Of these women, 1168 (87%) were successfully linked to the registries.

### Outcomes

For this study prespecified outcomes were the first occurrence of an acute cardiovascular event, either myocardial infarction or ischaemic stroke, whichever occurred first.

The outcome myocardial infarction includes the diagnoses defined by the ICD-9-CM codes 4100 to 4109 and the cause of death defined by the ICD-10 code I21. The outcome ischaemic stroke includes the diagnosis defined by the ICD-9-CM codes 433, 4330 to 4333, 4338, 4339, 434, 4340, 4341, and 4349 and codes 4350- to 4359 (transient ischaemic stroke), and the cause of death defined by the ICD-10 codes I63 and I64. We chose to include these two acute cardiovascular diseases, as these will most likely lead to hospitalization and therefore captured by the Dutch Hospital Data register.

### Statistical analyses

Follow-up ended on the date of a first incident cardiovascular event of interest, the date of death, or 31st December 2012, whichever came first. Median follow-up was calculated using the estimates of the censoring distribution for overall mortality.

To explore the influence of an increased coagulation propensity on the risk of a recurrent cardiovascular event we compiled an individual prothrombotic score (coagulation score). The score was constructed with coagulation markers measured in both patient groups and in control subjects, being: 1) tissue factor/tissue plasminogen activator induced clot-lysis time (CLT) as a measure of fibrinolytic potential [19]; 2) antigen levels of coagulation factors of the intrinsic coagulation system (factor XII, FXII, and FXI and prekallikrein) [20]; 3) inhibitor complexes of the serine proteases of the intrinsic coagulation system (C1 esterase inhibitor for FXIIa, FXIa, Kallikrein, and antitrypsin inhibitor) as measures of the activation of intrinsic coagulation factors [21]; 4) a disintegrin-like and metalloprotease with thrombospondin type 1 motif, member 13 (ADAMTS13) antigen levels [22]; 5) von Willebrand factor (VWF) antigen levels [22]; 6) antiphospholipid antibody tests (presence of lupus anticoagulant detected with dilute Russell’s viper venom time (dRVVT) reagents, IgG anticardiolipin antibody concentrations, IgG anti-β2-glycoprotein I concentrations and anti-prothrombin antibodies concentrations) [9]; 7) presence of factor V Leiden (either heterozygous or homozygous) [23, 24]; 8) presence of G20210A mutation in the prothrombin gene (either heterozygous or homozygous) [23, 24]; 9) presence of the homozygous form of the methylenetetrahydrofolate reductase (MTHFR) C677T [23, 24]. The score was based on the beta coefficients, adjusted for matching variables, obtained from logistic regression models for the association between each coagulation marker and index ischaemic stroke. These analyses were based on dichotomous exposures, with the 90th percentile of the control group distribution as cut off value, or on the presence of the genetic variant, or on the test positivity, whichever was the most appropriate. These beta coefficients were summed so that the compiled score represents the coagulation weighted prothrombotic potential for each patient, with higher values of the score corresponding to a higher levels of thrombotic propensity.

To investigate whether high values of the coagulation score increased the risk of a recurrent event, we applied a Cox proportional hazard model based on the quartiles of the prothrombotic score distribution as exposure categories, with the lowest quartile as reference. The model was adjusted for age, sex, body mass index, alcohol consumption, smoking history, diabetes mellitus, hypertension, hyperlipidaemia and family history of a cardiovascular event.

## Results

**Fig. 1:**
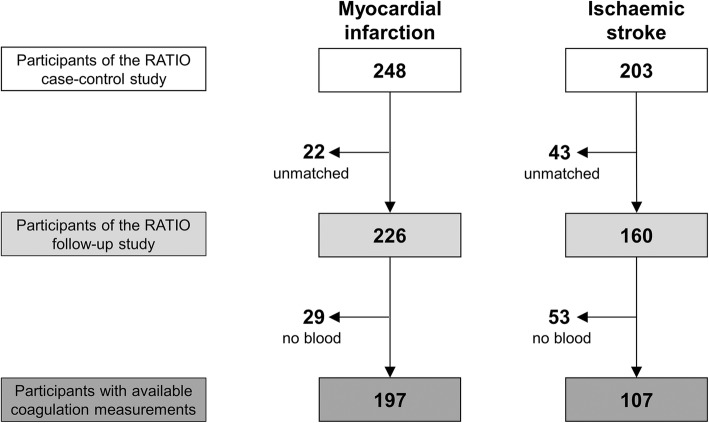
Flow chart for study participants

The RATIO follow-up study included 226 patients with myocardial infarction and 160 patients with ischemic stroke followed for a median time of 18.7 years (IQR 17.5–20.5). Their baseline characteristics are depicted in Table 1. The coagulation score could be calculated for 197 patients with myocardial infarction and 107 patients with ischaemic stroke, for whom blood and biomarker measurement were available (Fig. 1). Mean values of the score were 1.08 (min − 0.36, max 5.73) in patients with myocardial infarction and 2.06 (min − 0.28, max 9.03) in patients with ischaemic stroke.

**Table 1:** Baseline characteristics for patients included in the RATIO follow-up study

	Myocardial infarction *n* = 226	Ischaemic stroke *n* = 160
Demographic characteristics		
Age at the event, years (SD)	42.4 (6.1)	40.0 (7.5)
BMI, kg/m^2^ (SD)	27.1 (5.4)	25.5 (5.7)
Median follow-up, years (IQR)	18.6 (17.2–20.1)	18.9 (17.3–20.7)
Medical history, *n* (%)		
Diabetes mellitus	11 (5%)	9 (6%)
Hypertension	56 (25%)	41 (26%)
Hyperlipidaemia	25 (11%)	9 (6%)
Alcohol consumption	138 (61%)	90 (57%)
Smoking	188 (83%)	100 (63%)
Cardiovascular family history^a^	145 (66%)	82 (56%)

*BMI* body mass index, *SD* standard deviation, *IQR* interquartile range

^a^Data available for 97% of the subjects in the myocardial infarction group and 92% of the subjects in the ischaemic stroke group

**Fig. 2:**
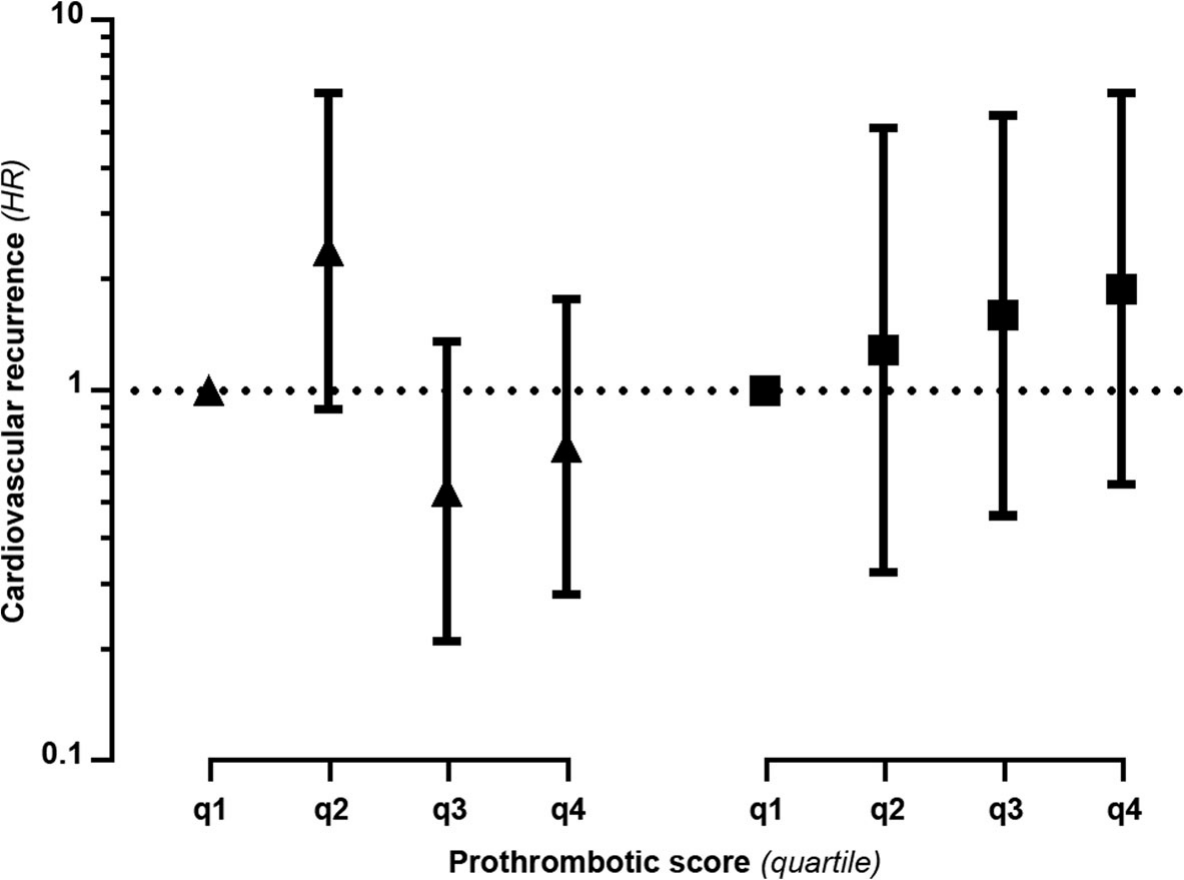
Hazard ratios for cardiovascular recurrence by quartiles of the coagulation score. Triangles indicate hazard ratios for cardiovascular recurrences in patients with myocardial infarction by quartile of the coagulation score, whereas squares indicate hazard ratios for cardiovascular recurrences in patients with ischaemic stroke by quartile of the coagulation score. Hazard ratios are obtained by Cox proportional hazard models and are all adjusted for age, smoking, alcohol consumption, BMI, history of diabetes, hypertension, and hypercholesterolemia and family history of a cardiovascular event. Solid lines indicate 95% confidence intervals. The y axis scale is logarithmic. q indicates quartile of the coagulation score. The first quartile (q1) is the reference category

During follow-up, 36 (18%) patients with myocardial infarction and 23 (21%) patients with ischaemic stroke experienced a recurrent event, of which 11 and 4, respectively, were fatal. Figure 2 shows the association between quartiles of the coagulation score and the risk of a recurrent cardiovascular event, with the first quartile as reference category. In patients with myocardial infarction high values of the coagulation score (i.e., high thrombotic propensity) were not associated with an increase in risk of a recurrent event compared with low values of the score (i.e., low thrombotic propensity; fourth quartile vs first quartile HR 0.7, 95% CI, 0.3–1.8). On the contrary, a doubling of the risk of cardiovascular recurrences was observed in patients with ischaemic stroke and high values of the coagulation score compared with patients with low values, although with a very wide confidence interval (fourth quartile vs first quartile HR 1.9, 95% CI 0.6–6.3), with evidence of a “dose response” relationship (second quartile vs first quartile HR 1.3, 95% CI, 0.3–5.1, and third quartile vs first quartile HR 1.6, 95% CI, 0.5–5.6).

## Discussion

Our findings suggest that an increased coagulation tendency is associated, in a dose dependent manner, with a decades long increased risk of cardiovascular recurrences in women with ischaemic stroke but not in women with myocardial infarction. Although women with ischaemic stroke and an overt cardiac-embolic-source were excluded from this study, all other subtypes were combined as data needed for classification were not available [25]. This hampers the ability to better elucidate the pathophysiological mechanisms beyond our observation. However, we believe our finding may have clinical relevance in the future, given its possible implications for secondary prevention, especially in the era of the direct oral anticoagulants (inhibitors of factor IIa, and factor Xa) or even newer anticoagulants such as FXI antisense oligonucleotides, that represent new treatment options to establish a more targeted anticoagulation. The direct clinical impact of our results is limited as we did not set out to develop a formal risk prediction model and therefore should also not be interpreted as such. Future studies, with a more homogenous subsample of stroke patients are needed to show the true added value of markers of hypercoagulability in terms of long-term risk prediction.

Patients enrolled in our study a few years after the event, and therefore, patients suffering from early fatal ischaemic stroke or myocardial infarction are not included in the analyses. This implies that our results should be applied to survivors of both ischaemic stroke and myocardial infarction. In fact, including patients from the moment of their stroke would mix long-term and short-term effects, potentially hindering their interpretation. The applied coagulation score included several acquired and inherited markers of hypercoagulability and was weighted on the risk of the index ischaemic stroke event, since index ischaemic stroke has been shown to be the arterial event in which hypercoagulability plays the largest role [10, 11]. In this way, the coagulation score represents the individual prothrombotic propensity. Even if our risk score does not include all markers of coagulation, we were still able to include 18 presumed markers of hypercoagulability as they were measured in both case groups of the RATIO study.

A possible limitation to our study is that recurrent arterial cardiovascular events (both myocardial infarction and ischaemic stroke) were not objectively confirmed, but obtained from a national register (the Dutch Hospital Data register). However, data collected in this register have been found to be reliable (for myocardial infarctions the percentage of corrected diagnosis was almost 100%) [18]. In addition, even if any misclassification was present in the register, it is likely to be non-differential, and therefore, the comparison between myocardial infarction and ischaemic stroke is unaffected. Another limitation is that, despite the long follow-up, numbers of recurrent arterial cardiovascular events were small, leading to imprecision of the estimated rates (wide confidence intervals). Moreover, we were unable to incorporate information on medication use during the follow-up period into our analyses. Some medications, such as anticoagulants, antiplatelets and statins may have influenced the prothrombotic score. However, the ischaemic stroke patients did not use anticoagulant as their stroke was of non-cardioembolic origin. Antiplatelets and statins at baseline have at most a marginal effect on measured coagulation factors, but given that prescription of these drugs as such is not dependent on the prothrombotic score, it is unlikely that this would have caused a strong bias. Finally, we cannot exclude that our results partly reflect a different involvement of inflammation in the relationship between hypercoagulability and recurrences. Unfortunately, we do not have data in order to investigate this hypothesis.

## Conclusions

Our results show that an increased coagulation tendency might be associated with long-term cardiovascular risk in women with ischaemic stroke but not in women with myocardial infarction.

## Abbreviations

BMI: Body mass index
HR: Hazard ratio
ICD: International Statistical Classification of Diseases and Related Health Problems
IQR: Interquartile range
RATIO: Risk of Arterial Thrombosis in Relation to Oral Contraceptives
SD: Standar deviation
